# Accrediting retail drug shops to strengthen Tanzania’s public health system: an ADDO case study

**DOI:** 10.1186/s40545-015-0044-4

**Published:** 2015-09-25

**Authors:** Edmund Rutta, Jafary Liana, Martha Embrey, Keith Johnson, Suleiman Kimatta, Richard Valimba, Rachel Lieber, Elizabeth Shekalaghe, Hiiti Sillo

**Affiliations:** Management Sciences for Health, Arlington, VA USA; Management Sciences for Health, Dar es Salaam, Tanzania; Pharmacy Council of Tanzania, Dar es Salaam, Tanzania; Tanzania Food and Drugs Authority, Dar es Salaam, Tanzania

**Keywords:** Private sector, Drug sellers, Accreditation, Medicines, Tanzania

## Abstract

**Introduction:**

Retail drug sellers are a major source of health care and medicines in many countries. In Tanzania, drug shops are widely used, particularly in rural and underserved areas. Previously, the shops were allowed to sell only over-the-counter medicines, but sellers who were untrained and unqualified often illegally sold prescription drugs of questionable quality.

**Case description:**

In 2003, we worked with Tanzania’s Ministry of Health and Social Welfare to develop a public-private partnership based on a holistic approach that builds the capacity of owners, dispensers, and institutions that regulate, own, or work in retail drug shops. For shop owners and dispensers, this was achieved by combining training, business incentives, supervision, and regulatory enforcement with efforts to increase client demand for and expectations of quality products and services. The accredited drug dispensing outlet (ADDO) program’s goal is to improve access to affordable, quality medicines and pharmaceutical services in retail drug outlets in rural or peri-urban areas with few or no registered pharmacies. The case study characterizes how the ADDO program achieved that goal based on the World Health Organization’s health system strengthening building blocks: 1) service delivery, 2) health workforce, 3) health information systems, 4) access to essential medicines, 5) financing, and 6) leadership and governance.

**Discussion and evaluation:**

The ADDO program has proven to be scalable, sustainable, and transferable: Tanzania has rolled out the program nationwide; the ADDO program has been institutionalized as part of the country’s health system; shops are profitable and meeting consumer demands; and the ADDO model has been adapted and implemented in Uganda and Liberia. The critical element that was essential to the ADDO program’s success is stakeholder engagement—the successful buy-in and sustained commitment came directly from the effort, time, and resources spent to fully connect with vital stakeholders at all levels.

**Conclusions:**

Beyond improving the quality of medicines and dispensing services, availability of essential medicines, and the regulatory system, the impact of a nationwide accredited drug seller approach on the pharmaceutical sector promises to provide a model framework for private-sector pharmaceutical delivery in the developing world that is sustainable without ongoing donor support.

## Background

Private medicine retailers are principal players in promoting access to medicines in low- and middle-income countries [1]. For many years, drug shops and pharmacies have been recognized for their potential to improve health across a wide area of illnesses and health issues [2, 3]. A review of literature looking at the role of drug sellers in child health in Africa reported that caretakers’ use of retail drug outlets for child illnesses ranged from 15 to 82 % with a median around 50 %, and that they used retail outlets even when cheaper alternatives existed, such as village health workers [4]. In addition, drug shops are popular with the poorest populations; in sub-Saharan Africa, for instance, 10.5 % of people in the lowest wealth quintile sought primary care from drug shops, compared to just 2.8 % of people in the highest wealth quintile [5]. Despite their popularity and potential, drug shops are not generally considered part of the larger health system and are mostly missing from countries’ health strategies, policies, and monitoring.

### *Duka la Dawa Baridi* in Tanzania

Prior to 2003, *duka la dawa baridi* constituted the largest network of licensed outlets for basic essential medicines in Tanzania. They were located in all of the country’s districts, and their combined inventory turnover value was estimated to be greater than Ministry of Health and Social Welfare (MOHSW) expenditures on essential medicines for primary health care [6]. Because full-service pharmacies are located almost exclusively in major urban areas (60–70 % percent in Dar es Salaam alone), while approximately 75 % of Tanzanians live in rural and peri-urban communities, *duka la dawa baridi* were often the most convenient drug outlet [7].

Although important as a source of medicines for a significant proportion of the population, data from a 2001 assessment [8] indicated that *duka la dawa baridi* were associated with problems that included—

- Questionable medicine quality
- Inadequate storage for medicines
- Untrained staff
- Inadequate regulatory enforcement and supervision
- Authorization to sell only a limited list of over-the-counter medicines
- Illegal dispensing of prescription medicines

### Developing a public-private accreditation program

The Tanzania Food and Drugs Authority (TFDA), the original program champion, and Management Sciences for Health, the technical partner, designed the accredited drug dispensing outlet (ADDO) program to address these problems. The program’s goal was to improve access to affordable, quality medicines and pharmaceutical services in retail drug outlets in rural or peri-urban areas with few or no registered pharmacies.

To achieve that goal, we took a holistic approach that combines changing the behavior and expectations of those who use, own, regulate, or work in retail drug shops. For shop owners and dispensing staff, this was achieved by combining training, incentives, supervision, and regulatory enforcement with efforts to affect customer demand for and expectations of quality products and services. Major program activities that contributed to this strategy included—

- Developing accreditation based on government-instituted standards and regulations
- Creating a public sector-based regulatory and inspection system and strengthening local regulatory processes and capacity
- Developing drug shop owners’ business skills
- Providing ADDO owners with business incentives, such as access to microfinancing and legal authorization to sell a limited list of essential prescription medicines, including some antimicrobials
- Facilitating access to convenient and reliable sources of quality medicines
- Changing behavior of dispensing staff through training and supervision
- Improving awareness of customers regarding quality and the importance of treatment compliance through marketing and public education

Early evaluation results provided evidence that ADDOs could improve access to quality pharmaceutical products and services, and that a fully regulated, comprehensive private sector pharmaceutical services system in Tanzania could have a substantial impact on health care [6, 7]. While acknowledging the significant costs and time needed for full national implementation, the MOHSW and TFDA were convinced that rolling out the ADDO program to all areas of the country was warranted and that the broad societal and health sector benefits justified the cost. Consequently, the MOHSW approved scale-up of the ADDO concept throughout mainland Tanzania and signaled its further embrace of the program by announcing phase-out of all unaccredited *duka la dawa baridi* by 2011.

The ADDO model is based on the assumption that to effectively and sustainably tackle the problem of access^1^ to quality medicines and pharmaceutical services in a resource-limited setting, all aspects of the drug shop enterprise—the physical premises, medicine inventory management, providers’ capacity and interactions with consumers, and appropriateness of recommended treatments according to national guidelines—must be addressed comprehensively and systematically. In addition, the larger pharmaceutical sector in which drug shops operate also needs to be strong and include product licensing and supply, training, record-keeping, reporting, and inspection.

### The role of drug shops in the health system

The ADDO program is an interesting example of how to use regulation “control knobs” to influence and structure the performance of the retail private pharmaceutical sector [9]. Because health systems vary widely depending on the context, a best-practices model needs to be adapted for local conditions. But health systems that function well share certain characteristics: they have procurement and distribution systems that actually deliver medicines to those in need; they are staffed with a sufficient number of skilled and motivated health workers; and they operate with financing systems that are sustainable, inclusive, and fair—the costs of health care should not force households into poverty [10]. However, in sub-Saharan Africa, most public health systems do not function effectively—the degree of which depending on factors such as political stability, health financing and infrastructure, management and oversight, and availability of qualified personnel and commodities. As a result, people commonly fill the void through the private sector, which has received relatively little attention by national and international policy makers.

## Case study

This case study addresses the key study question: Is the Tanzania ADDO model scalable, transferable, and sustainable?^2^ Specifically, it asks—

- Was the decentralized ADDO implementation model effective in scaling up the ADDO program nationally? What broader efforts helped to ensure scalability?
- Was Tanzania’s ADDO model effectively adapted for Ugandan and Liberian needs and replicated?
- Are accredited drug seller initiatives and their effects sustainable without donor support?

This case draws data and information from an archive of published and unpublished reports and studies including results from our own data collection related to the ADDO program. The initiative was piloted in the Ruvuma region starting in 2003 and by mid-2013 had been rolled out to every region in mainland Tanzania. The case study uses the World Health Organization’s six health system building blocks as a framework to characterize the ADDO program and its contributions: 1) service delivery, 2) health workforce, 3) health information systems, 4) access to essential medicines, 5) financing, and 6) leadership and governance.

### Service delivery

As the ADDO program took off, many recognized the potential of these shops to not only increase access to essential medicines, but also to serve as a platform for community-based public health interventions; for example, a child health training module for dispensers is based on the Integrated Management of Childhood Illness initiative and includes danger signs of pneumonia in children and the appropriate action, co-trimoxazole treatment or referral, depending on the situation [11]. Other programs that have been integrated include tuberculosis case identification and referrals, distribution of subsidized artemisinin-based combination therapies, and knowledge and control of antimicrobial resistance [12–14]. Other public health interventions that are being pursued include creating a system of referral linkages between community health workers, ADDOs, and health facilities relating to maternal and child health and family planning and the incorporation of rapid diagnostic testing for malaria into ADDO services.

**Fig. 1:**
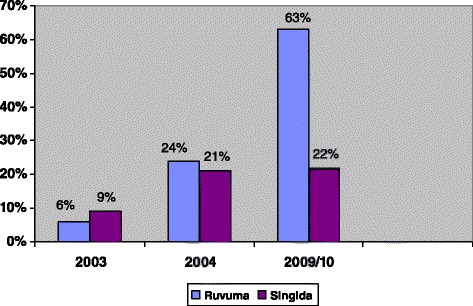
Percentage of encounters with appropriate malaria treatment in intervention and control regions: 2003–2010

As a result of interest in the concept, numerous organizations and programs played a role in expanding both the services that ADDOs provide and their geographic reach—over 9,000 accredited shops or shops close to being accredited are currently serving the 25 regions of mainland Tanzania. And improvements have been sustained: In 2010, 63 % of malaria encounters in Ruvuma were treated according to the treatment guidelines, compared to 24 % in 2004 (end of Ruvuma pilot), and 6 % before the ADDO program started—a 950 % improvement [15] (Fig. 1). In addition, 301 people taking part in a household survey in Ruvuma overall had a positive impression of ADDOs and ADDO services (Table 1).

**Table 1:** Household opinions of ADDOs in Ruvuma: 2010

Opinion	% (*n* = 301)
Obtains most medicines from ADDOs	86
ADDOs improve quality of care	84
Drug shop is clean and tidy	79
Attendant is knowledgeable	79
Medicines are available	76
Medicines are of good quality	68
Prices are affordable	46

### Health workforce

“This program complements the government efforts to ensure that the communities get access to medicine. It is more valid here in Namtumbo where public health facilities are few in number and the district is facing a real crisis of shortage of skilled health workers. I do not know what would have been the situation without the ADDOs. Our people depend on them” —District health official, Namtumbo district, Ruvuma region [16].

Increased efforts to fight HIV/AIDS, malaria, and tuberculosis in sub-Saharan Africa have accentuated the critical shortage of health care workers, including physicians, nurses, and pharmacists. According to the World Health Organization, Tanzania had about one pharmacy professional (pharmacist or pharmacy technician/assistant) for every 100,000 people at the time the ADDO program launched, with almost all working in urban areas [17]. Throughout Africa, programs have been testing innovative approaches to ease the health care personnel shortage [18, 19].

ADDO Dispenser Curriculum Topics

**Table:** 

• Laws, regulations, and ethics
• Good dispensing practices and rational medicines use
• Common medical conditions in the community
• Reproductive health and HIV/AIDS
• Communication skills and counseling
• Child health
• Record-keeping

Rutta and colleagues [16] described how the government of Tanzania addressed a lack of access to quality medicines and services by creating a new cadre of pharmaceutical service provider based in ADDOs. Although many interventions targeting retail drug sellers have been carried out in Africa and Asia, they usually have focused singularly on training or other capacity-building activities and have often been limited to a particular concern, such as malaria or child health [20, 21]. Because of its comprehensive approach to addressing public and private sector issues as well as consumer demand, the ADDO program not only fills a human resource niche in the health system, but can also be a career pathway for dispensers.

As the text box illustrates, in addition to appropriate dispensing, training also focuses on the importance of record-keeping. Owners and dispensers learn how to monitor product sales, keep track of expiry dates, and what types of products to stock to improve business. In a survey of owners and dispensers after accreditation, almost all reported keeping financial records (94 %) and monitoring daily sales (98 %); in addition, 69 % tracked monthly profits [22]. Although the program design included owners’ incentives to become accredited, such as an expanded list of allowable drugs to stock, in our surveys, owners have repeatedly mentioned dispenser training as the most highly valued program benefit. Tanzania now has over 20,000 dispensers trained with the government-approved curricula. This equates to approximately 43 trained personnel per 100,000 population.

Another important feature is the program’s impact on women—particularly rural women who have few employment options. By the time the last region was rolled out in mid-2013, approximately 90 % of ADDO dispensers and 39 % of ADDO owners were women. This program, therefore, provides a unique opportunity for many women to supplement their family’s income, while at the same time taking on a leadership role within their community.

### Health information systems

As mentioned, ADDO owners and dispensers are trained to keep records related to business and sales. Dispensers track who buys medicines (in addition to select demographic information) and for what conditions. The availability of such records at ADDOs has allowed supervision and inspection teams to review and assess ADDO dispensers’ performance and the shops’ compliance with regulations. The records provide information that could not only help health officials quickly identify problems, such as epidemics, but could also provide surveillance information on common conditions in the community. However, these data are generally not easily communicated and are therefore not reported to health authorities. In addition, the regulatory authority (now the Pharmacy Council^3^) needed more efficient ways to track activities related to ADDOs and pharmacies, such as premises location, inspection dates, license renewal, and accreditation status.

To help improve information access, we worked with a local technology firm, Invention and Technological Ideas Development Organization (ITIDO), to build a web-based regulatory database and website for the Pharmacy Council. The database uses unique identification numbers for ADDOs and pharmacies as well as for all personnel. ITIDO trained Pharmacy Council staff on maintenance and use of the database and handed over to them the necessary equipment. We also trained Pharmacy Council staff in how to use GPS devices to geo-code premises and Google Earth maps to locate the geo-coded ADDOs and pharmacies.

Our research had shown that most ADDO owners and dispensers have basic mobile phones. ITIDO developed mobile applications for payment, indicator reporting, and information exchange through an SMS-based helpline between ADDOs and Pharmacy Council that does not require smart phones. We piloted the full mobile package in Pwani region and the mobile payment component in Pwani and Dar es Salaam. Of 140 ADDOs in the pilot, an average of 80 % regularly reported on eight indicators, such as drug availability, using mobile phones from May through August, 2014. During that time, Pharmacy Council received 3,378 messages on the SMS-helpline and sent out 22,818 messages to ADDOs, pharmacies, and pharmacy professionals. Table 2 shows two months’ worth of data from Pwani region on ADDO service statistics on child health and family planning.

**Table 2:** Mobile phone-based indicator reporting in Pwani region (July–August 2014)

# Total clients attended	57,528
# Under 5 years attended	17,082
# Under 5 with malaria	9,138
# Under 5 with pneumonia	3,786
# Under 5 with diarrhea	3,792
# Under 5 referred	1,356
# Clients receiving oral contraceptive pills	7,464

Incorporating information technology including mobile money and other applications has a potentially major impact on drug seller initiatives’ sustainability. Dispensers have had no trouble reporting basic service statistics, and during the pilot, 129 people used mobile money to pay over 12 million TSH in fees. Shop personnel appreciate the ease of paying fees through M-Pesa: *“This system is good. In the past I had to close my shop and go all the way to the Council, and there you might find a long queue and sometimes you spend the whole day there”* (Dispenser, Kibaha) [23]. In addition, ADDO staff felt that it was much easier to get information through the new SMS-based helpline, although as the number of inquiries has increased, the Pharmacy Council is needing to develop and adhere to procedures for responding on a timely basis. Additionally, our evaluation showed that the Pharmacy Council required more coaching on how to take advantage of the information; although personnel knew how to access the data, it was not being used for decision-making. Orienting MOHSW officials to the newly available private sector data will likely increase demand for it.

### Access to essential medicines

For many common medical problems, such as malaria and diarrhea, a variety of factors lead people to self-diagnose and medicate before visiting a government health facility: distance to the health facility; lack of drug availability in the public facility; cash availability; and customer concerns about privacy and quality of the health care, facilities, and medicines [21, 24, 25].

#### Medicine quality

People were buying prescription-only medicines from drug shops in Tanzania long before shops were accredited, but they were being sold illicitly by untrained staff. Accreditation standards have broadened the list of medicines ADDOs can legally dispense to include basic essential medicines—both prescription and nonprescription—and required training on appropriate medicine use and referral. In addition, consumers who buy medicines in ADDOs can be assured that the quality is better because standards have improved drug storage and management practices. In our initial evaluation, we used drug registration status as a proxy indicator for the quality of drugs being sold in shops. After accreditation, the proportion of unregistered medicines in Ruvuma was reduced by a factor of 13, from 26 to 2 % [7]. In evaluations in other regions since then, no unregistered products have been found in ADDOs, and our recent research showed that 93 % of 243 drug samples passed quality tests [26]. ADDO dispensers also receive training on the importance of not selling drugs that have expired, and our research has indicated that expiry is no longer a major problem in the shops.

#### Medicine policies

The ADDO program has acted as a catalyst to drive the creation of new policies related to improving access to medicines in the private sector, including the 2009 MOHSW notice to phase out unaccredited drug shops by 2011, the establishment of the Medicine Access Steering Committee under the MOHSW to coordinate access initiatives in the private sector, and the National Malaria Control Program’s 2006 adoption of the ADDO platform as part of its strategy to increase access to malaria treatment, which paved the way for ADDOs to distribute subsidized artemisinin‐based combination therapy. Other significant policies include the incorporation of child health services into the ADDOs in 2006 and the National Health Insurance Fund decision to allow members to fill prescriptions at ADDOs in 2007. These pharmaceutical sector policies have strengthened Tanzanians’ access to essential medicines.

#### Medicine availability

As noted, one of the primary reasons that people choose ADDOs over health facilities is the more consistent availability of popular medicines. Accreditation increased the legal availability of some prescription-only medicines; in addition, our research has shown that ADDOs generally have better stock of medicines on hand for children. In our audit of 95 ADDOs and 72 health facilities in four regions in 2013, the availability of capsules and tablets in ADDOs and health facilities was similar, however, ADDOs had significantly better availability of suspensions and syrups, which are commonly prescribed to treat young children (p = 0.009).

ADDOs also continue to be included in strategies to increase community availability to treatment for childhood conditions. After neonatal causes, pneumonia (21 %) and diarrhea (17 %) are the leading causes of childhood mortality in Tanzania—both imminently treatable conditions [27]. In line with a number of relevant global initiatives, we have recently begun partnering with UNICEF to improve community access to new products to treat childhood pneumonia and diarrhea—amoxicillin dispersible tablets and oral rehydration solution (ORS)/zinc co-packs. UNICEF is working to increase access in the public sector, while our intervention will target 700 ADDOs in 12 districts to stock the two new commodities. Our activities include orienting owners and dispensers on how to manage and dispense the products and monitoring the availability through a mobile phone application.

### Financing

**Fig. 2:**
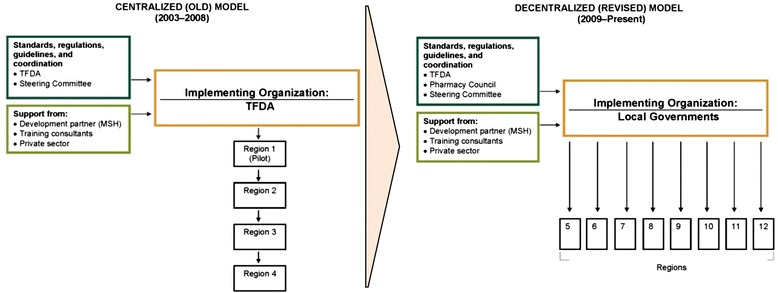
Evolution of ADDO implementation model in Tanzania

Developing and implementing an accredited drug seller initiative and bringing it to scale requires committed resources, individuals, and institutions. The initial funding for the conceptualization and pilot of the ADDO program in Ruvuma came from the Gates Foundation, but multiple stakeholders contributed to the program rollout and enhancement (Table 3). After the centrally implemented pilot program, we worked with stakeholders to decentralize implementation by using district-level teams that worked simultaneously in multiple regions, rather than having one national team covering one region at a time (Fig. 2). To further increase efficiency and reduce costs, the revised model’s dispenser training schedule was reduced from 45 to 30 days and shop mapping and preliminary inspection activities were merged. When the implementation model shifted responsibility from central to local authorities, the rollout time decreased from an estimated 18 months to less than 12 months per region, and TFDA records indicated that the estimated implementation cost fell by 55 % (126,000 USD per district compared with 57,000 USD per district).

**Table 3:** ADDO program milestones: 2003–2013

ADDO program phase	Year	Description
Assessment, program design, conceptualization and planning	2001–2003	The Strategies for Enhancing Access to Medicines (SEAM) Program, MOHSW, TFDA, and multisectoral stakeholders assessed access to essential medicines, recommended a public-private sector approach to improving access, and designed and reached a consensus on the ADDO model with Ruvuma as a pilot region. Program funding provided by the Bill & Melinda Gates Foundation.
Pilot program development and implementation—Ruvuma region	2003–2005	SEAM Program and TFDA design and launch the ADDO program in the Ruvuma region—210 outlets accredited. (Gates Foundation).
		Private sector contribution (210 owners’ investment for premises construction or upgrade to meet accreditation standards).
Pilot program M&E	2003–2005	SEAM commissioned monitoring and evaluation of the ADDO program in Ruvuma (Gates Foundation).
	2006	Danida sponsored an independent evaluation of the ADDO program in Ruvuma by HERA.
Program scale-up (centralized approach)	2006–2008	Government of Tanzania, through the MOHSW, approves TFDA plan to rollout ADDOs to Tanzanian mainland.
		U.S. Agency for International Development (USAID), through MSH’s Rational Pharmaceutical Management Plus Program, funds ADDO rollout in Morogoro region using resources from the President’s Emergency Plan for AIDS Relief—553 ADDOs.
		Government of Tanzania funds rollout in Mtwara and Rukwa regions—122 ADDOs.
		Private sector contribution (675 owners costs investment for premises construction or upgrade to meet accreditation standards) in Morogoro, Rukwa, and Mtwara.
	2007–2008	Danida supports TFDA to conduct training of trainers and district inspectors, develop and print training materials used for scale-up, and carry out national sensitization seminars with local governments.
Program scale-up (decentralized approach)	2007–2011	Gates Foundation funds the East African Drug Seller Initiative (EADSI) to work with TFDA to review and revise the existing ADDO model to make nationwide scale-up more cost-efficient and to help ensure the long-term sustainability of ADDOs and to evaluate effect of changes made on access to medicines and quality of products and services provided.
	2008	Tanzanian stakeholders agreed to decentralize implementation model to improve efficiency of scale-up and sustainability of program at consensus meetings in Dodoma and Morogoro.
	2008	Global Fund to Fight AIDS, Tuberculosis and Malaria agrees to fund ADDO rollout in six to eight high-impact malaria regions to improve access to antimalarials for children under five; Danida and government of Tanzania also contribute funding for rollout.
	2009	Clinton Health Access Initiative funds initial implementation activities in Shinyanga and Dodoma
	2008–2009	Local governments in Shinyanga, Tabora, Iringa, Arusha, Kagera, and Kilimanjaro took initiative on their own to mobilize funds to introduce ADDOs.
	2011	Cost of training in Dar es Salaam for the urban ADDO model funded by ADDO dispenser and owner contributions (~1,300 dispensers and ~1,700 owners).
	2013	Last region, Mwanza, launches the ADDO program in June 2013. Officially, ADDO program coverage is nationwide.
Program maintenance and sustainability; public health intervention integration into the ADDO program	2006	National Malaria Control Programme adopts the ADDO concept as part of its national strategy to increase access to malaria treatment.
	2006	MSH’s Rational Pharmaceutical Management Plus Program collaborates with the Basic Support for Institutionalizing Child Survival Project to add a child health component to ADDO services (USAID funded FY07, FY08, FY09).
	2007	Tanzania’s National Health Insurance Fund initiates plan that allows members to fill prescriptions at ADDOs.
	2007	MSH’s Strengthening Pharmaceutical Services Program uses President’s Malaria Initiative funds to provide subsidized artemisinin-based combination therapy through ADDOs (FY06, FY07, FY08).
	2008	The Prime Minister’s Office for Regional Administration and Local Government mandates local governments to incorporate ADDO program implementation into their planning and budgets.
	2009	Rockefeller Foundation funds MSH to develop a strategy to promote program sustainability and quality through the establishment of ADDO owner and dispenser associations.
	2009	Government of Tanzania regulation is revised to phase out unaccredited drug shops (*duka la dawa baridi*) by 2011.
	2011	Legislative change mandates the transition of program oversight from TFDA to Pharmacy Council.
	2010–2012	As a pilot, MSH’s Systems for Improved Access to Pharmaceuticals and Services Program collaborates with National TB and Leprosy Control Program to integrate interventions to engage 550 ADDOs in Morogoro to improve early detection of people with TB symptoms (USAID).
	2011–2015	Gates Foundation funds the Sustainable Drug Seller Initiative to ensure the maintenance and sustainability of these public-private drug seller initiatives in Tanzania and Uganda and to introduce and roll out the initiative in Liberia.

Once the ADDO program was established, the primary financing issue shifted to shop profitability and program maintenance—private sector shops need to stay in business and cover their costs and the public sector could not rely on ongoing donor contributions to fund program maintenance costs.

#### Shop profitability

From the start of the program, accredited shops have been profitable. Although the pilot program in Ruvuma linked the majority of owners with microfinance institutions to help them make the improvements needed to become accredited, few owners in other regions used that option to finance their shop renovations or operations. The majority of owners (79 %) reinvested their ADDO business profits; 11 % tapped personal savings for business financial needs; and 8 % used a combination of financing options [22]. Evidence of profitability also came from the willingness of owners and dispensers to take over all costs associated with accreditation, which had previously been donor funded. Additionally, when the National Health Insurance Fund incorporated ADDOs into its scheme, owners felt that the arrangement boosted their sales.

**Fig. 3:**
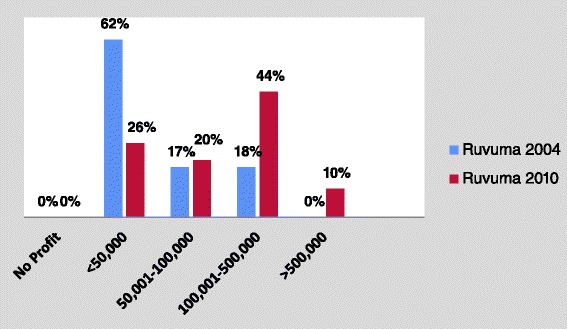
Average monthly net profit reported by ADDOs (TZS) in Ruvuma region: 2004–2010

We compared results of interviews with Ruvuma shop owners during our pilot’s endline evaluation in 2004 and then again in 2010. Those data show that none of the owners reported making no profit in either year (Fig. 3). Compared to 2004, where a majority of ADDOs (62 %) made a profit of less than 50,000 TZS per month, in the 2010 survey, only 26 % claimed that they made less than 50,000 TZS, while 44 % reported making a net monthly profit of 100,000 to 500,000 TZS which is two to ten times higher. Note that the value of the TZS was about 30 % less in 2004 compared with 2010 (~1,087 TZS to 1 USD in 2004 compared with ~1,430 TZS to 1 USD in 2010).

#### Program maintenance costs

In 2008 the government mandated local governments to include oversight of the ADDO program in their budgets. At the central level, ADDO maintenance costs are part of the annual budgets of both the Pharmacy Council and the TFDA. Government funding is supplemented by the annual license fees paid by pharmacies and ADDOs, which has increased along with program scale-up and as the Pharmacy Council has improved its ability to track payments using its database. The launch of the mobile money application also makes it easier for pharmacy and ADDO staff to pay fees to the Pharmacy Council.

#### Medicine sales prices

Our data have shown that average drug prices generally increase more in ADDO intervention regions immediately after implementation compared to control regions. This supports the assertion that ADDO owners tend to recoup some of their expenses by raising prices after incurring significant expenses in renovations, training, and inventory. To assess if the price increase is a temporary or permanent phenomena, we compared 2010 prices of select tracer items in Ruvuma to the 2004 prices by normalizing them with international prices. The average median price for a market basket of antibiotics compared with MSH’s *International Drug Price Indicator Guide* showed virtually no difference between 2004 and 2010 (+15 % compared with +16 %). Median prices in Ruvuma compared to the international price guide increased 1 % between 2004 and 2010 after adjusting for inflation. In addition, our recent research in four regions showed that the average price for a tracer list of medicines at 86 ADDOs was only 15 % more than the tracer list average at 98 public health facilities.

#### Institutionalized training

Stakeholders in Tanzania identified the institutionalization of training as a critical step toward independence from donor funding. In preparation for public and private training institutions to take over from the program (i.e., Management Sciences for Health and the national regulatory authority), we developed criteria for selecting training institutions and assessed the choices available in Tanzania and in Uganda, where we had also launched an accreditation program. We identified seven institutions in Tanzania—one for each zone: Lake, Northern, Central, Southern Highlands, Southern, Western, and Eastern. All seven have carried out ADDO dispenser training with costs ranging from 270,000 to 350,000 TZS per person (~150–200 USD). Separate courses and fees for business training are being implemented—this six-day business training costs in the range of 70,000 to 100,000 TZS per person (~40–56 USD). In Uganda, we selected two training institutions to deliver the first institutionalized seller and owner trainings, one located in an eastern district and one in a western district. Fort Portal International Nursing School completed its first training in March 2014 for 65 participants at a cost of 600,000 UGX (~240 USD) per participant. Since then, the school has trained 402 sellers in four additional districts in western Uganda. The school’s faculty delivered some of the drug seller courses within the districts themselves as opposed to holding them at their main campus in Fort Portal. This reduced travel costs and other expenses for the trainees and significantly increased course demand, which benefited the school financially.

Tanzania’s Pharmacy Council is responsible for the institutions’ training standards and oversight, and it issued a notice to all districts that ADDO trainings will now be conducted exclusively through approved training institutions, with ADDO owners and dispensers covering all costs. The Pharmaceutical Society of Uganda, which works with the National Council on Higher Education, now has responsibility for reviewing and updating the accredited drug shop curriculum as needed; it also accredits the training institutions.

### Leadership and governance

Development of the accreditation standards was a critical part of the model design that led to the overhaul of regulations governing *duka la dawa baridi* operations. TFDA technical committees reviewed and approved draft standards before the MOHSW approved them and the Minister signed them. Table 4 summarizes the standards for accreditation, which covers areas such as inspection. The development of the ADDO standards and code of ethics resulted from a comprehensive, wide-ranging stakeholder consultation process; nearly 400 people participated, including regional and district medical officers, members of Parliament, councilors, and *duka la dawa baridi* owners. The law requires all ADDO owners and dispensers to have a thorough understanding of these standards and ethics.^4^ It was amended in 2009 to accommodate the revision of the model to reflect decentralized implementation and operations—most notably, inspections.^5^

**Table 4:** ADDO program standards for accreditation

Component	Process or requirements
Accreditation application process	A Council Food and Drug Committee is responsible for a four-part application process for shops: an application form, initial inspection of the existing facility, re-inspection after any premise upgrades required for accreditation, and ongoing inspection after accreditation.
Incentives for owners	Owner incentives focus on improved shop profitability and approval to sell a range of prescription medications. Incentives for owners who commit to standards include access to micro-financing for stock purchases, a marketing campaign encouraging consumers to buy medicines at ADDO, and more reliable sources of affordable, quality wholesale goods.
Premises infrastructure	The standards provide instructions for building size, layout, identification, dispensing and services areas, storage, and security.
Staff qualification	The grade levels of ADDO dispensers include nurses, nurse-midwives, clinical officers, assistant medical officers, pharmaceutical assistants, and pharmaceutical technicians. The most common qualification of ADDO dispensers prior to ADDO training is nurse assistant.
Training	All dispensers must be accredited by the TFDA, display their accreditation certificate, and have their photo identification on their coats when working. Accreditation involves completing a TFDA-approved dispensers’ course. Course topics include in-depth information on ADDO drugs in their generic and brand forms; illness indications and contraindications; drug dosages, side effects, and patient information; laws governing dispensers’ work; basic management, record-keeping, and business ethics; and communications skills. ADDO training for shop owners focuses on ethics, regulations, and improvement of business management skills.
Drug quality and availability	The ADDO list of approved pharmaceuticals includes a full range of over-the-counter drugs and a limited list of prescription drugs, including common antibiotics and oral contraceptives. ADDOs may sell only those drugs registered with and approved by the TFDA. ADDO-restricted wholesalers can receive a license to sell nonprescription and ADDO-restricted approved prescription drugs under the supervision of a full-time pharmaceutical wholesaler’s technician.
Record keeping	ADDOs must keep records of all prescription drugs sold and their selling prices, financial and sales information, customer complaints, and expired medications. These records may be used for supervision purposes and must be available for review by inspectors.
Regulation, inspection, and sanctions	Local government officials receive a basic inspection training course from the TFDA and are certified as local inspectors. They work with the TFDA to conduct a minimum of two inspections of each shop annually. The program also carries out inspections of remaining unaccredited shops, and can issue sanctions against those that illegally sell prescription drugs. A channel exists for registering any customers’ complaints against ADDOs or any shops’ complaints about harassment by inspectors or other problems.

#### Local oversight

As the program expanded, it quickly became clear that a centralized TFDA did not have the capacity to continue implementing the rollout in new regions while overseeing the existing program. Therefore, as mentioned, an important aspect of revising the model was to decentralize the implementation and oversight responsibility to the district level. Although a national-level champion is key to a successful model launch, a mature program relies on the commitment of local officials to support inspection and program maintenance; consequently, 3,262 district and ward inspectors have been trained as part of the initiative.

#### Empowering provider associations

In addition to the government’s role in overseeing ADDO operations, we worked to strengthen existing professional drug shop provider associations and facilitate the formation of new provider associations to serve as a governance resource and a professional “voice” for the owners and dispensers. This work produced an association toolkit that was developed through a consultative stakeholder workshop [28]. It includes operational and management tools (available in English and Kiswahili) related to how to form and register and association, mobilize financial resources, and monitor and evaluate activities.

After a national mapping exercise identified 35 associations, we chose four model districts to serve as learning grounds (Bagamoyo, Kilombero, Mbinga, and Mbarali). After orientation workshops, each association received support to develop a three-year activity plan. Some of these activities included peer-peer supervision (Mbarali), income-generating activities such as land purchase (Mbarali), and establishment of savings and credit societies (Bagamoyo). As association services have increased, membership has grown and membership fees collected have doubled.

To evaluate this work, we interviewed owners and dispensers on their perceptions of how membership in an association can help them individually. ADDO owners said they expect the associations to—

- Give them a strong unified voice on matters relating to their businesses
- Help them access loans to improve their businesses
- Enable them to have joint procurement of drugs and other pharmaceutical products and enjoy the economies of scale resulting from bulk purchases
- Provide them with a platform to engage with various authorities such as Pharmacy Council, Tanzania Revenue Authority, and local government authorities
- Create a forum for them to share experiences and resolve conflicts among members
- Enable them to pool resources to start their own savings and credit cooperative societies and advance loans to members
- Provide them with a mechanism for self-regulation to minimize noncompliance with pharmaceutical sector regulations and standards

Likewise, dispensers mentioned that the associations will—

- Provide them with a platform to deliberate on issues of interest
- Give them a common voice to air grievances to owners
- Help them demand better salaries and work conditions, including standard working hours, overtime payment, and annual leave
- Provide them with a forum to exchange ideas and experiences in line with their training and enable them to improve their skills and promote self-compliance to regulations
- Enable them to pool resources and invest in other income-generating activities toward a goal of individual development and self-improvement

#### Peer supervision

A challenge with the accreditation model has been providing regular supportive supervision to dispensers. While local officials periodically inspect shops to ensure compliance with standards, they do not have the time or resources to make more frequent supervisory visits. Therefore, a notable activity was the development of association-based peer-peer supervision in Ugandan Accredited Drug Shops (ADS) in Mityana district. Since the peer supervision started in October 2013, peers who were nominated by their associations have made supervisory visits every two to three months. In turn, those peer supervisors received support from the association leadership and the regional Pharmaceutical Society of Uganda representative and technical team. Peer supervisors helped the ADS sellers with issues related to taking steps to improve their performance, rational medicine use, use of rapid diagnostic tests, patient referral, quantification of supplies, dispensing equipment, and self-assessments.

A pre/post evaluation of Mityana with Kyenjojo district serving as a control found that ADS in both districts experienced improvements in various indicators including management of common conditions, dispensing practices, and product availability. However, Table 5 shows Mityana’s more robust results related to management of simple diarrhea in children. We see peer supervision organized through provider associations as a sustainable way to complement local government supervision and inspection.

**Table 5:** Peer supervision effect on management of non-bloody diarrhea in Ugandan children

Category of children	Mityana before	Mityana after	Kyenjonjo before	Kyenjonjo after
Presented with non-bloody diarrhea	129	348	62	21
Received zinc and ORS	83/129 (64 %)	330/348 (95 %)	42/62 (68 %)	16/21 (76 %)
Received oral antibiotics	27/129 (21 %)	23/348 (7 %)	29/62 (47 %)	12/21 (57 %)
Referred	6	1	2	0

#### Introducing and growing health-related consumer advocacy

Also in Uganda, we collaborated with the Coalition for Health Promotion and Social Development (HEPS) to create and implement a consumer advocacy strategy related to expectations about pharmaceutical services and rights regarding health care. HEPS implemented the strategy in Kamuli district by training community leaders and outreach volunteers to attend community events to talk about the public’s health rights, what services the public should expect from drug shops, and rational medicine use. As a result, awareness of medicines issues in the community increased. *“The community members now ask the drug sellers important questions about medicine packaging and handling which was not there before,”* one volunteer in Nawanyago said [29]. The district community development officer heard many local leaders caution people on medicines use at meetings he attended in villages and parishes. Community cognizance about drug shops also resulted in changes; for example, after receiving public pressure, an unqualified drug shop owner switched from selling medicines to selling clothes in Balawoli sub-county, and in Kitayunjwa, a general merchandise shop quit selling medicines. HEPS has been approached to carry out similar consumer advocacy initiatives in other districts that other donors will fund.

## Discussion and evaluation

"One thing is certain, informal providers represent a growing and undeniable force within the health community, and despite the refusal of some governments and others to acknowledge/engage with them, it is in all our interests—particularly the patients—to begin working more effectively with these elusive actors"—Tom Feeny, HANSHEP [30].

**Fig. 4:**
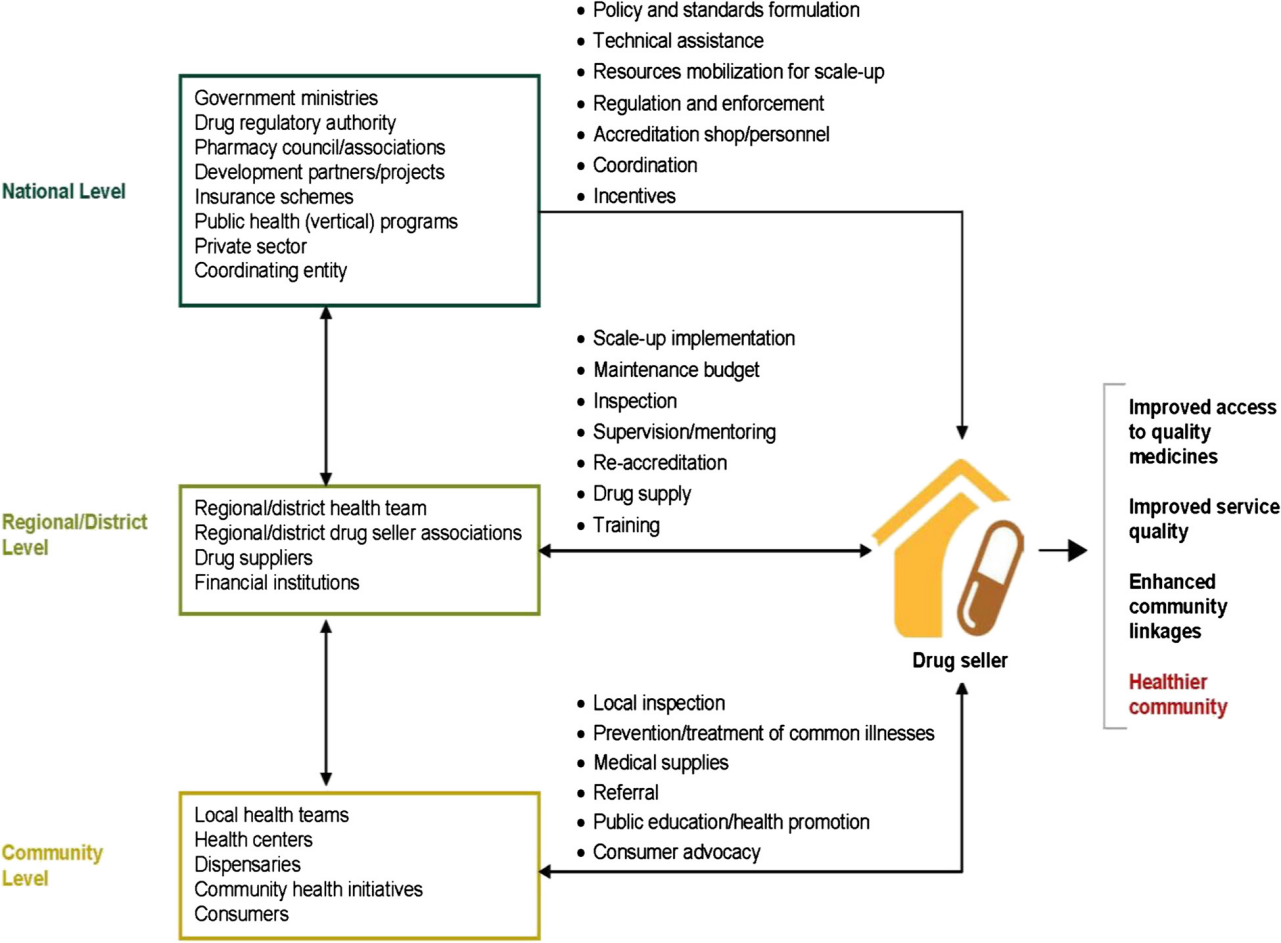
Accredited drug seller initiative conceptual framework

The goal of the ADDO program has been to improve access to affordable, quality medicines and pharmaceutical services in retail drug outlets in rural or peri-urban areas where there are few or no registered pharmacies. To achieve this goal, the ADDO model took a holistic approach that combines developing the capacity of owners, dispensers, and institutions that regulate or work in retail drug shops. For shop owners and dispensing staff, this has been achieved by combining training, incentives, supervision, and regulatory enforcement with efforts to affect client demand for and expectations of quality products and services. Figure 4 illustrates the drug seller initiative conceptual framework.

### Stakeholder engagement: the linchpin of success

The primary element that has been essential to the ADDO program’s success is stakeholder engagement—the successful buy-in and sustained commitment came directly from the effort, time, and resources spent to fully connect with all vital stakeholders at all levels. Involvement ranged from publicly stating support for the concept to working closely on all aspects of the program design and implementation. At the end of the pilot in Ruvuma, regional and district stakeholders reported the following program strengths: use of a participatory approach that involved all stakeholders from the beginning: owners, dispensers, consumers, political leaders; a fair and transparent process for permit application and approvals; the dispenser training component; and respecting and valuing community-level inputs [7].

During the program’s critical early period, TFDA in collaboration with MOHSW organized a study visit of members of Parliament’s Social Welfare Committee to ADDOs in Ruvuma. Their enthusiastic reaction paved the way for the allocation of additional funding for rollout from the government. In her budget presentation to the Parliament in 2004, the Minister of Health, Hon. Anna Abdallah, reported that 1.06 million USD had been allocated to expand the ADDO program following the promising results from the pilot in Ruvuma. Such a high-level commitment of the government’s own budget provided an incentive for continued donor interest and support, which is illustrated in the list of program milestones (Table 3). Another sign of government ownership was the various strategic documents that started addressing the ADDO program as a “key MOHSW program” and not a “donor-funded project.” For example in the Ministry’s *Health Sector Strategic Plan III July 2009–June 2015: Partnership for Delivering the MDGs*, the ADDO program was cited as fundamental in the private sector provision of essential medicines to vulnerable groups [31, 32].

The purpose of this case report was to answer questions related to the scalability, transferability, and sustainability of the Tanzania ADDO model specifically, but also the accredited drug seller initiative in general.

### Scalability of the Tanzanian ADDO model

Funded by the government of Tanzania and multiple development partners over a decade-plus period, Tanzania’s ADDO program achieved nationwide scale-up in June 2013. The time needed to mobilize the funding and regulatory capacity delayed scale-up from the government’s original 2011 target. From the proof-of-concept pilot in Ruvuma region in 2003 through scale-up, the ADDO program evolved to address public health needs and economic realities. As mentioned, the estimated implementation cost decreased by 55 % from decentralization and incorporating efficiencies in training; in addition, as owners and dispensers realized the benefits of accreditation, they became more willing to pay for accreditation costs, such as branding, renovations, increased inventories, and training that had been covered by donors in the program’s early years. The shift of implementation to the districts from the central level also allowed quicker scale-up because multiple regions could be scaled up simultaneously—while it took Tanzania six years to roll out the ADDO program in four regions under the original centralized model, 10 more regions completed implementation within three years using the decentralized approach.

### Transferability of the ADDO model to other country contexts

We have demonstrated the extent to which the ADDO model can be transferred and replicated in other countries’ settings through the establishment of ADS in Uganda and Accredited Medicine Stores (AMS) in Liberia. Management Sciences for Health worked with national and local stakeholders to develop an accreditation model based on the Tanzanian experiences, but adapted to the two countries’ different needs and health and pharmaceutical sectors. Liberia, especially, offered a unique opportunity because of its status as a post conflict state and our shift of focus to an urban rather than rural setting.

As of July 2015 in Uganda and Liberia, 689 and 313 shops, respectively, were accredited or awaiting accreditation, and 1,330 sellers had been trained in Uganda and 664 in Liberia. Both countries have developed scale-up strategies that will phase out the current class of unaccredited shops. Liberia also changed its regulations to formalize AMS as part of Liberia’s health care delivery system. In both countries, evaluations showed that ADS and AMS increased the availability of quality pharmaceutical products and improved dispensing and business skills. For example, the percentage of ADS offering injections, which are illegal in drug shops in Uganda, fell from 74 % to 0 after accreditation in the pilot district. In Liberia, the percentage of expired, damaged, or counterfeit products on the shelves of drug shops went from 28 % at baseline to 8 % at endline, while the percentage of owners keeping business records rose from 33 % at baseline to 80 to 90 % over three monitoring periods after the intervention.

### Sustainability of the ADDO model

Our goal was to ensure the maintenance and sustainability of public-private drug seller initiatives that increase access to and appropriate use of quality pharmaceutical products and services for common conditions in underserved populations. Several model components focus specifically on increasing efficiency and thereby contributing to program sustainability and independence from donor funding. They include the following—

- Incorporating technology, including mobile money and SMS-based reporting, to facilitate payments and communication between the shops and the regulatory body
- Strengthening shop owner/dispenser associations to empower providers to govern themselves through peer-supervision and create mechanisms to boost members’ financial sustainability
- Empowering the community to increase demand for quality pharmaceutical services and products
- Institutionalizing training by shifting the responsibility and costs from the public to the private sector

## Conclusions

Using the World Health Organization’s health system building blocks as a framework, this case study demonstrates that the creation of a public-private partnership using government accreditation to increase access to quality pharmaceutical products and services in underserved areas of Tanzania was scalable, sustainable, and transferable. The primary means to success was stakeholder buy-in and ownership—the country must drive the activity, not the donor or the organization providing technical support. However, implementing an accredited drug seller program is complex, with political, legal, regulatory, professional practice, and economic challenges to address. Consequently, developing an initiative and bringing it to scale is an expensive venture. If limited funding is available and used to complete only discrete pieces of what is needed to create a sustainable whole, then failure can result if interest wanes or the situation changes before all pieces can be completed.

Through our initial work in Tanzania and then in Uganda and Liberia, we have expanded access to medicines and treatment, but also solidified the global view that public-private initiatives to strengthen the quality of pharmaceutical products and services provided by retail drug sellers are feasible and effective in multiple settings. The impact of the ADDO approach on the pharmaceutical sector—and on society as a whole—promises to provide a model framework for private-sector pharmaceutical delivery in the developing world.

## Footnotes

1 We define access to medicines by physical availability, affordability, geographic accessibility, and acceptability (or satisfaction).

2 Scalable defined as the ability to expand an intervention to support larger system without affecting performance; transferable defined as extent to which an intervention can be replicated and implemented in a new country; sustainable defined as persistence of the intervention’s effect over time.

3 Until 2011, TFDA was the regulatory authority responsible for ADDOs; however, a regulatory change placed the responsibility with the Pharmacy Council.

4 The Tanzania Food, Drug and Cosmetics Act (standard and code of Ethics for Duka la Dawa Muhimu Regulation 2004.

5 The Tanzania Food, Drug and Cosmetics Act (standard and code of Ethics for Duka la Dawa Muhimu (Amendments) Regulation 2009.
