# Erratum to: Accrediting retail drug shops to strengthen Tanzania’s public health system: an ADDO case study

**DOI:** 10.1186/s40545-015-0049-z

**Published:** 2015-11-19

**Authors:** Edmund Rutta, Jafary Liana, Martha Embrey, Keith Johnson, Suleiman Kimatta, Richard Valimba, Rachel Lieber, Elizabeth Shekalaghe, Hiiti Sillo

**Affiliations:** Management Sciences for Health, Arlington, VA USA; Management Sciences for Health, Dar es Salaam, Tanzania; Pharmacy Council of Tanzania, Dar es Salaam, Tanzania; Tanzania Food and Drugs Authority, Dar es Salaam, Tanzania

Following publication of the original version [1] of the article in the Journal of Pharmaceutical Policy and Practice, it was brought to our attention that the Alliance for Health Policy and Systems Research’s support is missing in the acknowledgement section.

The authors would like to add the following text:

“The authors would like to acknowledge the support of the Alliance for Health Policy and Systems Research (HSR/HIS), World Health Organization which supported the development of this case study and publication of this work.”
